# CH/π Interactions in Carbohydrate Recognition

**DOI:** 10.3390/molecules22071038

**Published:** 2017-06-23

**Authors:** Vojtěch Spiwok

**Affiliations:** Department of Biochemistry and Microbiology, University of Chemistry and Technology, Prague, Technická 3, Prague 6, 166 28, Czech Republic; spiwokv@vscht.cz; Tel.: +420-22044-3028; Fax: +420-22044-5140

**Keywords:** carbohydrate-protein interactions, CH/π interactions, lectins, interaction energy, non-canonical hydrogen bond

## Abstract

Many carbohydrate-binding proteins contain aromatic amino acid residues in their binding sites. These residues interact with carbohydrates in a stacking geometry via CH/π interactions. These interactions can be found in carbohydrate-binding proteins, including lectins, enzymes and carbohydrate transporters. Besides this, many non-protein aromatic molecules (natural as well as artificial) can bind saccharides using these interactions. Recent computational and experimental studies have shown that carbohydrate–aromatic CH/π interactions are dispersion interactions, tuned by electrostatics and partially stabilized by a hydrophobic effect in solvated systems.

## 1. Carbohydrate–Aromatic Interactions

**Figure 1:**
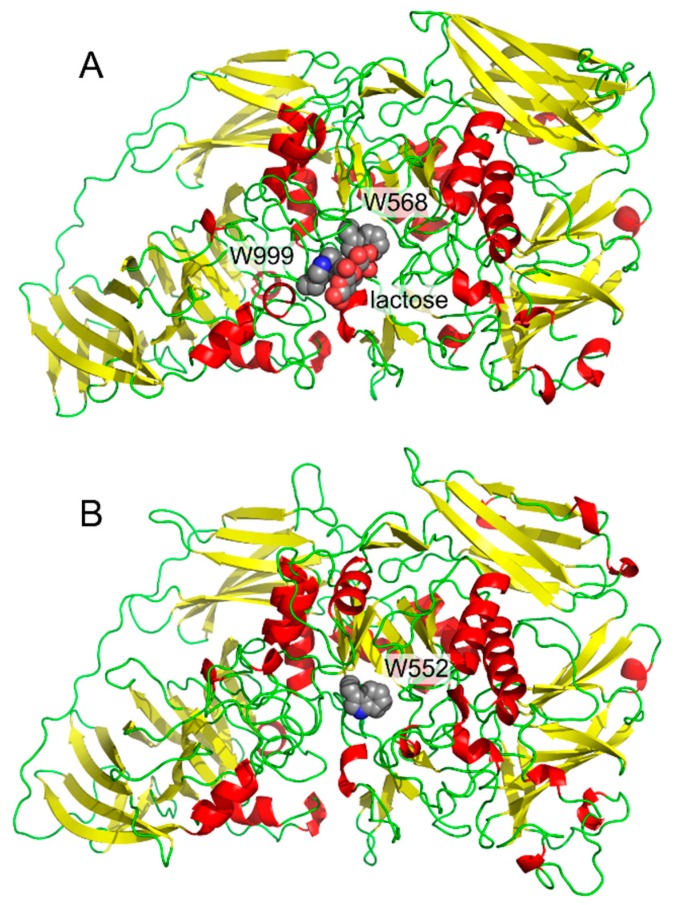
Comparison of β-galactosidases monomers from *E. coli* (**A**, with bound lactose, PDB ID: 1JZ8) and *Arthrobacter* sp. C2-2 (**B**, PDB ID: 1YQ2).

Our first contact with interactions between carbohydrates and aromatic systems was in the project focused on cold-active enzymes. We isolated the gene [1], cloned [1], recombinantly produced [1], and in collaboration with crystallographers, determined the three-dimensional structure [2,3] of a cold-active β-galactosidase from Antarctic bacterium *Arthrobacter* sp. C2-2. The project was focused on structural features of cold-active enzymes, thus we compared its structure with a mesophilic (normal temperature loving) enzyme. β-Galactosidase from *E. coli* [4] was a natural choice as the mesophilic counterpart. The presence of the residue Trp999 in *E. coli* enzyme and the absence of the corresponding tryptophan residue in the cold-active enzyme (Figure 1) was the most striking difference in active sites of both enzymes. This residue in *E. coli* β-galactosidase forms a parallel (“stacking”) interaction with bound substrate [3,4]. This residue had also been previously shown by site-directed mutagenesis experiments to be important in catalysis and transglycosylation reactions (alternative transferase reactions found in many glycosidases) [5]. Later, it turned out that unique catalytic properties of the cold-active enzyme could not be attributed to this single residue; nevertheless, the residue Trp999 in *E. coli* β-galactosidase inspired our research for several years.

A closer look at this type of interaction has shown that these are widespread in carbohydrate recognition [6,7,8]. To illustrate this, it is possible to statistically evaluate the presence of residues in the vicinity of carbohydrate moieties in experimentally determined three-dimensional protein structures. The Glyvicinity tool from the glycosciences.de package [9] can be used for this purpose. It can be shown that tryptophan is the most abundant amino acid residue (12.1%) in the vicinity of β-d-glucopyranose in experimentally determined protein structures (distance < 5 Å, resolution < 3 Å, 9 April 2017). Furthermore, this number is likely to be negatively biased by non-specific complexes, with glycosidic detergents used to facilitate protein crystallization. The fact that tryptophan is the most abundant residue in the vicinity of β-d-glucopyranose is in contrast with the fact that this residue is the least abundant in human proteome (1.3%, based on codon usage [10]). More detailed analysis can be found in the work of Hudson et al. [11].

Tyrosine is the second most abundant residue in the vicinity of β-d-glucopyranose with 9.0%, despite also being a rather non-frequent amino acid in proteins. A similar, but not so strong, trend can be observed for other monosaccharide residues found in common glycoconjugates.

**Figure 2:**
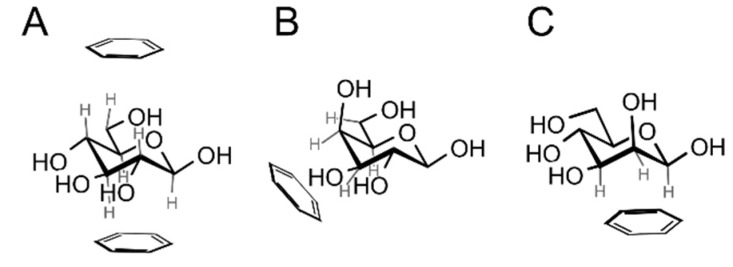
Prevalent geometries of carbohydrate–aromatic CH/π interactions: (**A**) β-d-glucopyranose can interact via both faces (only the *A* face is available for α-d-glucopyranose); (**B**) α- or β-d-galacto- or d-fucopyranose interacts via hydrogens on atoms *C3*, *C4*, *C5* and *C6* (a mirror assembly is typical for l-fucopyranose); (**C**) β-d-mannopyranose interacts via hydrogen atoms on *C1*, *C2* and *C3*.

β-d-Glucopyranose is structurally predisposed for carbohydrate-aromatic interactions thanks to the fact that all of its ring C–H hydrogens are oriented axially. This makes it possible to interact with an aromatic system (indole of Trp or benzene of Phe or Tyr) in a parallel stacking geometry (Figure 2A), either at the *A* (top) or *B* (bottom) face of the carbohydrate ring (see the work of Rose et al. [12] for definition of faces; the *B* face corresponds to the α face in the anomer-based terminology in the d-hexopyranose series). α-d-Glucopyranose favors only the *A* face due to the fact that the anomeric hydroxyl group blocks the *B* face. Another common monosaccharide readily interacting with aromatic residues is α- or β-d-galactopyranose or α-l-fucopyranose. These carbohydrates interact via a patch of C–H groups at carbons *C3*, *C4*, *C5* and *C6* at the *B* face (Figure 2B). Similarly, β-d-mannopyranose can form a similar patch by C–H groups of carbons *C1*, *C2* and *C3* (Figure 2C). We can speculate that hexopyranoses found in common glycoconjugates were evolutionary selected from 16 possible stereoisomers for their ability to form carbohydrate–aromatic interactions. However, it must be mentioned that other carbohydrate-related factors [13] may be involved in the evolutionary selection of monosaccharides [14].

**Figure 3:**
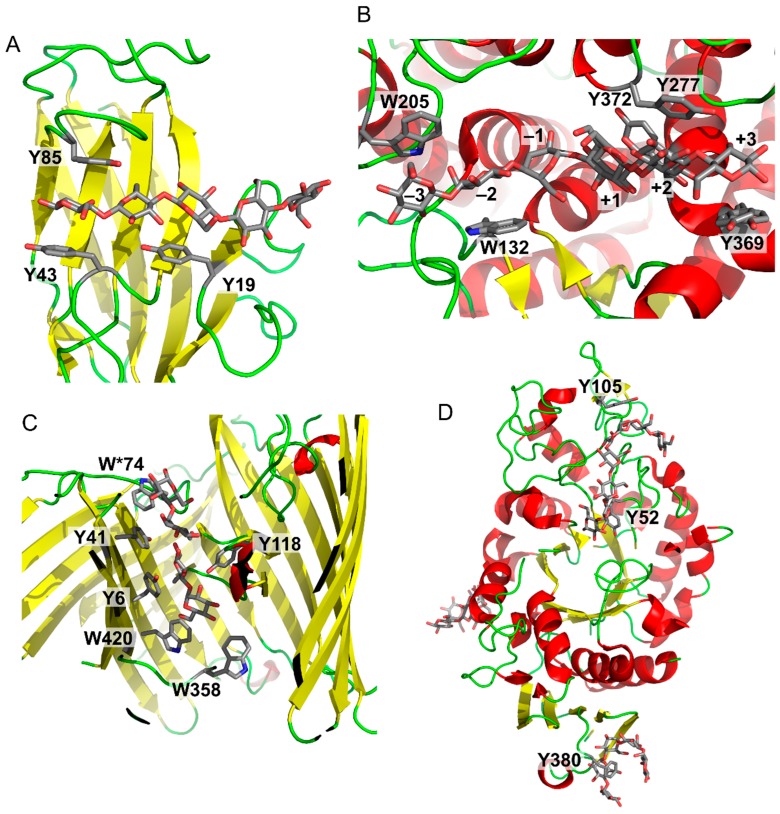
Examples of carbohydrate–aromatic complexes. (**A**) *Cellulomonas fimi* carbohydrate binding module CMB4 with (β-d-Glcp)_5_ (PDB ID: 1GU3); (**B**) *Clostridium thermocellum* endoglucanase CelA with (β-d-Glcp)_6_ (PDB ID: 1KWF); (**C**) “Greasy slide” of *E. coli* LamB maltoporin with two maltose molecules (PDB ID: 1MPM, residue W*74 belongs to another protein chain); (**D**) “Sugar tongs” in barley α-amylase (Y105 and Y52 form the active site; Y380 forms the “sugar tongs” site).

Figure 3 shows a series of examples of carbohydrate–aromatic interactions in natural systems. They show that carbohydrate–aromatic interactions are involved in a wide range of processes including carbohydrate binding, catalytic processing and transport. This overview can start with a carbohydrate-binding protein, in particular a lectin. As mentioned above, β-d-glucopyranose can form carbohydrate–aromatic interactions by either *A* or *B* face. Occasionally it forms a complex by both faces, as illustrated by carbohydrate binding module CBM4 from *Cellulomonas fimi* [15] (Figure 3A). Carbohydrate processing enzymes are represented by endoglucanase from *Clostridium thermocellum* [16]. The structure of an inactive mutant of this enzyme depicted in Figure 3B shows a hexasaccharide chain bound to the enzyme. Five of these six monosaccharide residues interact by carbohydrate–aromatic residues; two with tryptophan and three with tyrosine residues. The only exception is the catalytic-1 site (other sites are binding sites). Other examples of enzymes cleaving β-d-glucopyranosides indicate that carbohydrate–aromatic interactions (in the orientation depicted in Figure 2A) are not compatible with catalysis. On the other hand, the orientation of d-galacto (and also d/l-fuco) depicted in Figure 2B is compatible with catalysis (possibly due to a longer distance from the cleaved bonds) as illustrated by *E. coli* and *Arthrobacter* β-galactosidase (Figure 1). A similar geometry to β-d-galactosidase can be found in a complex of cholera toxin with its carbohydrate ligand, which is one of strongest carbohydrate–protein complexes known [17].

An interesting aromatic system involved in carbohydrate recognition and transport is “greasy slide” found in maltoporin [18] (Figure 3C). This carbohydrate transporter contains an aromatic patch formed by two tryptophans and two tyrosines on one side, and another tyrosine at the opposite side of the transport tunnel. The transported maltose can “slide” on these aromatic surfaces into the cell. Mutagenesis studies demonstrated an important role of these residues for the transport function [19].

Another interesting example of carbohydrate–aromatic interactions are “sugar tongs” [20] (Figure 3D). It was found that barley α-amylase 1 contains a surface tyrosine residue (Y380) distant from the active site. Despite its high distance from the active site its mutation to a non-aromatic residue drastically decreases affinity towards its substrate and its catalytic activity [21]. It is supposed that it helps the enzyme to interact with starch substrate as supported by a site directed mutagenesis and structural biology studies [20,21].

**Figure 4:**
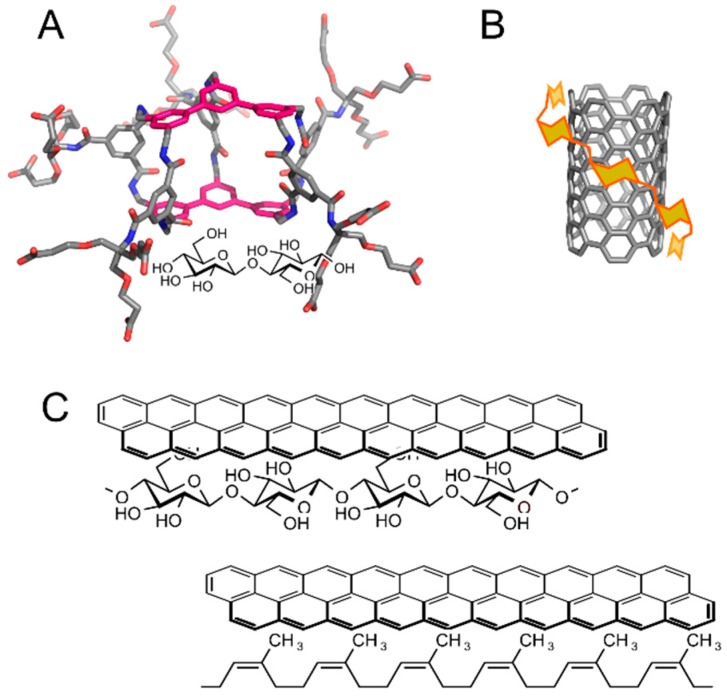
Examples of artificial carbohydrate–aromatic complexes: (**A**) An artificial carbohydrate receptor with ligand cellobiose (illustrative 3D structure generated by Avogadro [25]); (**B**) Schematic view of carbon nanotube solubilization by amylose; (**C**) Writing by pencil on a paper (top) and erasing it by a pencil eraser (bottom).

A cellulose–lignin assembly represents an example of natural carbohydrate–aromatic interaction between a carbohydrate and a non-protein system [22]. Carbohydrate–aromatic interactions can be found also in fully artificial systems. Artificial carbohydrate receptors are being developed for various applications, including future sugar sensors or pathogen scavengers. Many artificial carbohydrate receptors with different chemotypes have been developed [23]. Notably, receptors involving aromatic systems in carbohydrate–aromatic stacking geometries are highly successful. Davies et al. synthesized a series of carbohydrate receptors with an example shown in Figure 4A [24]. In water it binds cellobiose to its cavity with very high affinity in comparison with other artificial receptors (*Kd* = 1–2 mM) and with remarkable specificity.

There are many other examples of artificial systems indicating an important role of carbohydrate–aromatic interactions in molecular recognition. For example, porous graphitic carbon columns can be successfully applied in the chromatography of carbohydrates, despite their high polarity [26]. Carbohydrates were also successfully used in the solubilisation of carbon-based nanomaterials such as carbon nanotubes [27,28] (Figure 4B). In addition, carbohydrate–aromatic interactions can be exploited in formation of gels from low-molecular weight compounds, such as FMOC-(fluorenylmethylcarbonyl-) or napthylmethylcarbonyl-functionalised gluco- or galactosamine [29].

The most lovely example of carbohydrate–aromatic interaction is writing with a pencil on paper, first coined by Motohiro Nishio in his book about CH/π interactions [30] (Figure 4C). A pencil contains aromatic molecules of graphite. Paper is composed of cellulose, which is a β-d-glucan with highly exposed C–H bonds (Figure 4C, top). A trace made by a pencil on a paper can hardly be washed away by polar as well as non-polar solvents, but it can be erased by a rubber eraser (Figure 4C, bottom), which is a competitive donor of C–H bonds.

## 2. Physical Nature of Carbohydrate–Aromatic Interactions—CH/π Bond

Noncovalent interactions can be classified based on geometry (e.g., stacking interactions), interacting moieties (e.g., hydrogen bonds, cation–π or halogen bonds) or based on their physical nature (e.g., electrostatic or dispersion). Carbohydrate–aromatic interactions are often seen as stacking interactions due to parallel orientation of the interacting carbohydrate and aromatic rings and also as CH/π interaction due to the fact that C–H bonds typically point toward the aromatic system.

The physical nature of carbohydrate–aromatic interactions was not clear when we started to study them. There were three different explanations found in the literature, all of them driven by chemical intuition rather than by experimental or computational results. The first group of authors explained carbohydrate–aromatic interactions as mostly electrostatic interactions between a partially negatively charged center of the aromatic ring and a partially positively charged hydrogen atom on a C–H bond (or more precisely, an aromatic quadrupole and a polarized C–H bond). The second group of authors explained these interactions as an example of CH/π interactions of mostly disperse nature. The third group of authors saw these interactions as purely hydrophobic, i.e., the effect of solvation and desolvation processes.

In order to elucidate the physical nature of these interactions we [31,32,33,34] and other authors [35,36,37,38,39,40] carried out a series of quantum chemical calculations. In principle, energy stabilizing in an intermolecular complex can be calculated as a difference between energy of the complex and energies of both interacting molecules. The quantum chemistry method must be carefully chosen and corrections to possible artifacts must be applied [41]. The value of energy stabilizing certain a noncovalent interaction can be further dissected into various components, most notably electrostatic, polarization and dispersion. Precise dissection into these terms require application of special computational methods (reviewed by Phipps et al. [42]). However, contribution of these two components can be roughly estimated from energies calculated by different quantum chemistry methods. Electrostatic interactions are, in general, well addressed by uncorrelated and density functional theory (DFT) methods. In contrast, uncorrelated and DFT methods usually fail in modeling of interactions of dispersion nature and correlated *ab initio* methods must be used. In the other words, molecular complexes dominated by electrostatic interactions are modeled as attractive by both correlated and uncorrelated methods, whereas interactions of dispersion nature are modeled as attractive only by correlated methods. Uncorrelated and most DFT methods underestimate the strength of dispersion interactions. This fact can be used to elucidate the nature of the bonds.

Quantum chemical energies of interactions [31,32,33,34,35,36,37,38,39,40] in different systems shaped our view of carbohydrate–aromatic interactions. First, these interaction are surprisingly strong, i.e., comparable in terms of energy with classical hydrogen bonds. Second, these interactions are of dispersion nature with minor role of electrostatics. It supports the theory of dispersion CH/π interactions. Finally, these results are rather neutral in relation to the hydrophobic theory. It shows that hydrophobic interactions (mediated by solvation and desolvation processes) are not necessary for binding, i.e., these interactions can be stable enough even in vacuum. However, hydrophobic interaction can contribute and further strengthen interactions in real carbohydrate–protein complexes.

These results of quantum chemical calculations were later supported by experimental spectroscopic studies [43]. Screen et al. used an IR ion-dip (IRID) technique setup involving formation of a molecular beam containing a carbohydrate (methyl- or phenyl- α-d-gluco-, -galacto- and α-l-fucopyranosides and α-l-fucose) and an aromatic molecule (toluene), irradiation by IR and UV laser and mass spectrometry. This setup makes it possible to obtain a conformation-resolved precise (gas phase) IR spectra of carbohydrate–aromatic complexes. They have shown that, for example, α-l-fucopyranose forms a stable CH/π complex with toluene even in a gas phase, i.e., without solvation and desolvation processes. This is an experimental evidence showing strength of carbohydrate–aromatic CH/π interactions. It clearly demonstrates that these interactions are not (solely) hydrophobic.

The majority of quantum chemistry studies in the field used geometries of carbohydrate–aromatic complexes derived from experimentally determined structures. In these studies, the monosaccharide moiety was usually taken directly from the experimental structure containing a monosaccharide ligand (or “dissected” from oligo-saccharide or glycoside). The aromatic amino acid residue was also taken from the experimental structure and typically represented as a side chain (i.e., as 3-methylindole or cresol for tryptophan or tyrosine, respectively). The geometry of such a complex was optimized by a suitable quantum chemistry method (while keeping overall orientation of both moieties) and then the stabilizing energy was calculated at the best available level of theory. However, this approach lacks a systematic evaluation of many possible carbohydrate–aromatic orientations. For this reason we carried out a systematic scanning of energy surface characterizing these interactions [33]. Energy of each point on the surface was calculated by placement of a benzene molecule onto one of the faces of β-d-glucopyranose, β-d-mannopyranose or α-l-fucopyranose. The geometry of such a complex (3757 for each face and saccharide) was optimized using density functional tight binding with a dispersion correction (DFTB-D) while restraining the center of benzene in the starting position. Energy of the final pose was re-calculated by DFT with a dispersion correction (DFT-D). Selected minima were then evaluated using coupled cluster with single, double and perturbative triple excitations (CCSD(T)). These results support the previously observed strength of these interactions (stabilized by 3.54–5.40 kcal/mol) [33]. They also showed that these interactions are weakly directional, i.e., comparable energies were obtained for different positions of benzene relative to the carbohydrate.

Carbohydrate–aromatic interactions are often modeled by biomolecular simulations using molecular mechanics force fields. The question arises how these force fields perform in this task. In order to test this we compared energies calculated by force fields and quantum chemistry [32]. Surprisingly, molecular mechanics force fields (OPLS-AA, GROMOS, CSFF/CHARMM and Glycam/AMBER) performed well with good correlation between quantum mechanics and molecular mechanics levels of theory. The only exceptions were special force fields not intended for routine use in biomolecular simulations.

Experimental IRID studies have shown that carbohydrate–aromatic interactions are stable enough to exist in vacuum. However, this finding does not rule out the possibility that hydrophobic effect contributes to stability of solvated carbohydrate–protein complexes. Pioneer computational studies on thermodynamics of carbohydrate–aromatic interactions on isolated moieties were done by Wohlert et al. [44]. In order to test this on a carbohydrate–protein complex, we carried out a series of molecular dynamics simulations of hevein domain HEV32 with β-d-GlcNAc trisaccharide [45]. This system is very interesting for its small size, which makes it possible to simulate relatively long time scales [46]. It is also an excellent object for NMR studies [47]. Beside the simulation of this complex under real conditions, we also carried out a series of simulations under hypothetical conditions of absence or weakening of selected interactions. The method of molecular dynamics simulation makes it possible to simulate systems under hypothetical conditions not possible in a real experiment. Černý et al. developed this approach to demonstrate the role of dispersion interactions in stability of DNA and proteins [48,49]. We simulated the complex of HEV32 with trisaccharide and in these simulations we weakened selected classical (OH/O) hydrogen bonds and/or carbohydrate–aromatic (CH/π) interactions. It was necessary to weaken both types of interactions simultaneously to completely destabilize the studied complex. Separate weakening of a single type of interactions was not sufficient and the complex was relatively stable through the whole simulation.

There are two possible scenarios in dynamics of an artificially destabilized complex. Either, the complex is stabilized solely by physical (electrostatic or dispersion) interaction. Then, its simulation with weakened interaction is likely to lead to destabilisation and dissociation. In contrast, a complex stabilized mostly by hydrophobic (i.e., solvation/desolvation processes) can stay stable even when physical interactions are weakened. In our simulations we observed something between these two scenarios, which indicates that carbohydrate–aromatic interactions combine CH/π and hydrophobic interactions.

Experimental determination of these contributions became possible thanks to development of a technique based on Enhanced Aromatic Sequons (described later in this review). Chen et al. [50] analyzed selected carbohydrate–aromatic interactions and observed comparable stabilizing contribution of CH/π and hydrophobic interactions. Analysed carbohydrate–aromatic complexes were stabilized by free energy of 0.8–1.0 kcal/mol. CH/π interactions contributed by 42–72% (also stabilization by an intrinsic effect of glycosylation was observed in some complexes). These results completed the picture of carbohydrate–aromatic interactions also experimentally.

Another elegant approach used in the research of carbohydrate–aromatic interactions is dynamic combinatorial chemistry. The concept of dynamic chemistry is based on dynamic bonds, such as disulphides or Schiff bases. Asensio et al. studied reactions of molecules D-L-NH_2_ with O=CH-L’-A, where D are donors and A are acceptors of carbohydrate–aromatic interactions and L and L’ are suitable linkers [51,52,53]. The process of Schiff bases formation in a pool of donor or acceptor molecules is driven purely thermodynamically due to the dynamic nature of the bond. Populations of different products are driven by stabilities of interactions between donors D and acceptors A. Then, the equilibrium can be frozen by reduction of Schiff bases to corresponding secondary amines, which makes it possible to analyze the products. Asensio et al. used NMR, which makes it possible to determine not only composition but also to study the interactions between donors and acceptors.

These studies highlighted that the strength of carbohydrate–aromatic interactions depends strongly on the number of CH/π contacts [51,53]. They also showed that axial substituents may decrease stability by blocking the interacting phase (in contrast, axial -OH or -OCH_3_ groups on the opposite face stabilize carbohydrate–aromatic interactions) [53]. Equatorial -OCH_3_ moieties in methylglycosides contribute to strength of carbohydrate–aromatic interactions as they interact by CH/π interactions [53]. O/π interactions formed by ring oxygen are significantly weaker than CH/π interactions [53].

The most interesting results of dynamic combinatorial chemistry studies are related to the contribution of electrostatics. First, it was demonstrated that polarization of a CH group by attachment of an oxygen atom (ring oxygen or hydroxyl) stabilizes the complex by approximately 0.3 kcal/mol per activated C–H bond [52]. The strong role of electrostatics was also observed when aromatic acceptors were varied [53]. In order to rationalize these results, Asensio et al. calculated energies stabilizing corresponding complexes by quantum chemical methods and decomposed them into electrostatic, polarization and dispersive contributions (desolvation effect was also evaluated). They found that electrostatic (approx. 3–5 kcal/mol) and polarization (approx. 1–1.5 kcal/mol) quantum chemical energies strongly correlated with experimental stabilities (relative free energies of carbohydrate–aromatic complexes). The dispersion energy stabilizing these interactions was generally stronger (approx. 7–8 kcal/mol), but there was very weak correlation with experimental stabilities. This shows that dispersion interactions provide the main “glue” of carbohydrate–aromatic binding with very little variations caused by directionality or the electrostatic nature of donors and acceptors. On the other hand, electrostatic interactions are slightly weaker but they can further “tune” strength and the geometry of carbohydrate–aromatic complexes, because they are highly influenced by directionality and charge distribution on donor and acceptor molecules. This is also in agreement with the findings of recent bioinformatics study that highlighted the role of electrostatics in orientation and strengths of carbohydrate–aromatic complexes [11].

## 3. Applications

Understanding the physics of carbohydrate–aromatic interactions can be used in many applications. The above mentioned artificial carbohydrate receptor [23,24] is a prominent example of such applications. Another interesting application of carbohydrate–aromatic interactions is in Enhanced Aromatic Sequons [54]. Proteins can be *N*-glycosylated at asparagine residues of **N**XT or **N**XS motif. By systematic studies of glycoproteins the group of Jeffrey W. Kelly found that glycosylation of the motif FX**N**XT provides faster folding and higher stability of the corresponding glycoproteins. This can be explained by existence of carbohydrate–aromatic interactions between the phenylalanine residue and the *N*-acetylglucosamine residue attached to asparagine [55]. This motif was installed into various proteins by site-directed mutagenesis and successfully improved their stability [54].

Besides this, we must mention bioinformatic algorithms aimed at recognition of carbohydrate-binding sites in proteins on the basis of an amino acid sequence or 3D structure. For example, Doxey et al. [56] used a statistical approach to predict carbohydrate binding sites on a series of proteins. Among them, the tobacco pathogenesis-related protein PR-5d was selected and interaction with cellulose was demonstrated experimentally [56]. Aromatic amino acid residues played a major role in this prediction.

A direct application of knowledge of carbohydrate–aromatic CH/π interactions can be demonstrated on carbohydrate–protein docking. Carbohydrate docking is very difficult for a number of reasons, including generally weak binding, number of water-mediated interactions and pseudosymmetry of sugars. Involvement of CH/π interactions was proposed as another pitfall. This lead to development of the SLICK (Sugar–Lectin Interactions and doCKing) scoring function (i.e., structure-free energy relationship) tailored for carbohydrate docking [57]. This scoring function contains an explicit term for CH/π interactions in its equation. This method has been shown in combination with BALLDock to outperform general protein–ligand docking packages in carbohydrate–protein docking tasks.

## 4. Conclusions

Theoretical and experimental studies have formed our current view of carbohydrate–aromatic interactions. These interactions are widespread in carbohydrate–protein complexes. They are found in carbohydrate-binding proteins, carbohydrate-processing enzymes and carbohydrate transporters. They can be also found in complexes of carbohydrates with aromatic non-protein binding partners, natural as well as artificial. These interactions are examples of CH/π non-canonical hydrogen bonds. Carbohydrate–aromatic interactions can exist in vacuum without a hydrophobic effect, but this effect contributes to binding in natural carbohydrate–protein complexes. Understanding the nature and geometries of these interactions may be employed in searching for carbohydrate binding sites, docking of carbohydrates, design of carbohydrate receptors and other applications.
